# Medical Reports on the State of Health in His Majesty's Ship Venerable, in the Years 1814 and 1815, Employed in the West Indies, as Flag Ship of Rear Admiral Sir Phillip Durham

**Published:** 1818-02

**Authors:** John Forbes

**Affiliations:** formerly Surgeon of the Venerable


[To face Page 93.]

### GENERAL TABULAR VIEW OF DISEASES

### In His Majesty's Ship Venerable, in 1814, 1815.—Crew, 600 Men

| DISEASES. | 1814. | | | | | | | | 1815. | | | | | | | | TOTAL. | | | |
|---|---|---|---|---|---|---|---|---|---|---|---|---|---|---|---|---|---|---|---|---|
| | May | June | July | August | Sept. | Oct. | Nov. | Dec. | Jan. | Feb. | March | April | May | June | July | Aug. | Ent. on Sick List | Died | Invalid.* | Cured |
| Continued Fever | 6 | 4 | 3 | 6 | 0 | 4 | 4 | 2 | 2 | 1 | 0 | 11 | 13 | 3 | 6 | 3 | 68 | 1 | 4 | 63 |
| Dysentery and Diarrhœa | 1 | 6 | 2 | 9 | 3 | 6 | 2 | 4 | 2 | 1 | 5 | 10 | 3 | 5 | 6 | 7 | 72 | 3 | 2 | 67 |
| Hepatitis, Splenitis, &c. | 3 | 3 | 2 | 0 | 0 | 1 | 2 | 1 | 3 | 1 | 1 | 0 | 0 | 3 | 6 | 0 | 26 | 1 | 4 | 21 |
| Phthisis Pulmonalis | 0 | 2 | 1 | 1 | 2 | 0 | 1 | 2 | 0 | 2 | 1 | 0 | 1 | 1 | 3 | 0 | 17 | 2 | 7 | 8 |
| Pneumonia | 0 | 0 | 0 | 0 | 0 | 1 | 0 | 0 | 0 | 0 | 0 | 0 | 0 | 2 | 0 | 0 | 3 | 2 | 0 | 1 |
| Cholera | 0 | 1 | 0 | 1 | 0 | 0 | 0 | 0 | 0 | 0 | 0 | 1 | 1 | 0 | 0 | 0 | 4 | 0 | 0 | 4 |
| Catarrh } † | 4 | 1 | 1 | 3 | 1 | 4 | 5 | 0 | 1 | 0 | 1 | 3 | 0 | 2 | 5 | 5 | 36 | 1 | 0 | 35 |
| Rheumatism } † | 2 | 0 | 1 | 4 | 2 | 2 | 2 | 2 | 1 | 0 | 1 | 5 | 5 | 2 | 2 | 0 | 31 | 0 | 0 | 31 |
| Colica Pictonum | 0 | 0 | 0 | 0 | 0 | 0 | 1 | 0 | 5 | 3 | 0 | 1 | 0 | 0 | 1 | 2 | 13 | 0 | 0 | 13 |
| Erysipelas | 1 | 2 | 0 | 2 | 0 | 1 | 0 | 0 | 1 | 2 | 1 | 1 | 1 | 0 | 0 | 0 | 12 | 1 | 0 | 11 |
| Ophthalmia | 1 | 1 | 0 | 3 | 4 | 1 | 1 | 1 | 1 | 0 | 0 | 0 | 1 | 1 | 0 | 1 | 16 | 0 | 1 | 15 |
| Hydrothorax | 0 | 0 | 0 | 0 | 0 | 0 | 1 | 0 | 0 | 0 | 0 | 0 | 0 | 0 | 0 | 0 | 1 | 1 | 0 | 0 |
| Icterus | 0 | 0 | 0 | 0 | 1 | 0 | 0 | 0 | 0 | 0 | 0 | 0 | 0 | 0 | 0 | 0 | 1 | 0 | 0 | 1 |
| Cutaneous Eruptions | 0 | 0 | 0 | 0 | 1 | 1 | 1 | 0 | 1 | 0 | 0 | 0 | 0 | 0 | 1 | 0 | 5 | 0 | 0 | 5 |
| Mania‡ | 0 | 1 | 1 | 0 | 0 | 0 | 1 | 0 | 1 | 0 | 0 | 0 | 1 | 0 | 1 | 0 | 6 | 0 | 2 | 4 |
| Fish Poison | 0 | 0 | 0 | 3 | 3 | 1 | 0 | 0 | 0 | 0 | 0 | 0 | 0 | 0 | 0 | 0 | 7 | 0 | 0 | 7 |
| Constipation | 1 | 0 | 0 | 0 | 0 | 0 | 0 | 0 | 0 | 0 | 0 | 0 | 0 | 0 | 0 | 0 | 1 | 0 | 0 | 1 |
| Convulsions | 0 | 1 | 0 | 0 | 0 | 0 | 0 | 0 | 0 | 0 | 0 | 0 | 0 | 0 | 0 | 0 | 1 | 0 | 0 | 1 |
| Psora | 2 | 0 | 0 | 0 | 0 | 0 | 0 | 0 | 0 | 0 | 0 | 0 | 0 | 0 | 0 | 0 | 2 | 0 | 0 | 2 |
| Phlegmon | 9 | 6 | 13 | 7 | 8 | 11 | 2 | 1 | 9 | 5 | 5 | 6 | 3 | 3 | 6 | 7 | 101 | 0 | 0 | 101 |
| Otitis | 0 | 0 | 0 | 0 | 1 | 0 | 0 | 0 | 0 | 0 | 0 | 0 | 0 | 0 | 0 | 0 | 1 | 0 | 0 | 1 |
| Nephralgia | 0 | 0 | 0 | 0 | 0 | 0 | 1 | 0 | 0 | 0 | 0 | 0 | 0 | 0 | 0 | 0 | 1 | 0 | 1 | 0 |
| Nostalgia | 0 | 0 | 0 | 0 | 0 | 0 | 1 | 0 | 0 | 0 | 0 | 0 | 0 | 0 | 0 | 0 | 1 | 0 | 1 | 0 |
| Lues Venerea | 6 | 2 | 2 | 0 | 0 | 2 | 1 | 3 | 1 | 5 | 2 | 0 | 8 | 3 | 1 | 2 | 38 | 0 | 2 | 36 |
| Anomalous | 0 | 3 | 1 | 2 | 2 | 0 | 0 | 4 | 0 | 0 | 4 | 1 | 0 | 0 | 0 | 0 | 17 | 0 | 0 | 17 |
| Ulcers | 3 | 4 | 2 | 0 | 0 | 0 | 0 | 0 | 1 | 0 | 2 | 3 | 1 | 0 | 0 | 2 | 18 | 0 | 1 | 17 |
| Wounds and Accidents | 8 | 12 | 8 | 22 | 8 | 14 | 11 | 7 | 9 | 3 | 10 | 15 | 8 | 6 | 11 | 23 | 175 | 2 | 1 | 172 |
| TOTAL | 47 | 49 | 37 | 63 | 36 | 49 | 37 | 27 | 38 | 23 | 33 | 57 | 46 | 31 | 49 | 52 | 674 | 14 | 26 | 634 |

* In this Column are included those Cases sent to England for cure, or sent there because supposed incurable.

† These were all well marked and acute cases.

‡ This Column contains two relapses of the same case.

THE

**Medico-Chirurgical**

**JOURNAL AND REVIEW.**

VOL. V.] FEBRUARY, 1818. [NO. 26.

### PART I.

### ORIGINAL COMMUNICATIONS.

——— quid utile ———

MEDICAL REPORTS *on the State of Health in His Majesty's Ship* VENERABLE, *in the Years* 1814 *and* 1815, *employed in the* WEST INDIES, *as Flag Ship of* REAR ADMIRAL SIR PHILLIP DURHAM. By JOHN FORBES, M. D. formerly Surgeon of the Venerable.

THE following Reports of the state of health of the Venerable, during part of the period that ship was employed on the Leeward Island station, are transcribed verbatim (with the exception of the few notes which have been added during the transcription) from the official returns transmitted, every two months, to the Transport Board; the only alteration being in the tabular view of diseases prefixed, which has been constructed by simply throwing the different original tables into one. Of the very various circumstances relative to the production, history, and treatment of the different diseases that affected the crew of the Venerable while in the West Indies, I possess very copious and minute accounts; and, had I time and inclination to arrange these, they would form a communication very different, in interest and value, from the present meagre sketches. Considered, however, as a faithful, though unfinished outline of the history of health of six hundred individuals, suddenly removed from a northern to a tropical climate, and placed in circumstances particularly favourable for correct and minute observation, I am inclined to believe that even these, with all their imperfections, are not undeserving the attention of the medical naturalist. Under this impression, I have transcribed them from my common-place book, and now transmit them to the Editors, with the view of having them perpetuated in the pages of their Journal.

---

The Venerable was commissioned in 1808, and was principally employed in the seas adjoining the northern and western shores of France until her departure for the West Indies, in January 1814. During this time there was nothing particular in the state of health of her company. While in the West Indies, the most particular and unremitted attention was paid to the cleanliness and purity of the ship by all the officers, and particularly by my friend, Captain Parr, the first Lieutenant.

### First Report—for May and June, 1814.

The general mildness of the diseases noted in the table affords little room for remark. Of the five cases marked hepatitis, only one was a true inflammation of the liver—the others being derangements of the functions, rather than structure, of that organ. The cases of fever have all ultimately done well, and—whether we look to their symptoms or duration—must be considered as of a very mild kind. One was an old case of intermittent; another was a febrile accession, accompanied by an external local erysipelatous affection; and of the remaining eight, only four were well-marked cases of fever. Even these, whether from the very powerful means of cure employed in their commencement, viz. very copious blood-letting and purging,—or from their particular character—were, in their duration at least, truly ephemeral. In fact, fever is but little known at present on this station; and, when it occurs, death, or even danger, seems little apprehended. Ulcers, properly so called, and Dysentery, are equally unknown.

### Second Report—for July and August, 1814.

Of the period embraced by the preceding table of diseases, the Venerable was about four weeks at sea; and the remainder, moored in the harbour of the Saintes.*

* A cluster of small islands between Gaudaloupe and Dominique.

The weather has not varied much, having been, in general, fine and moderate, with the exception of some few days of high winds and heavy rains. Showers, indeed, have fallen on nearly one half of the days, but they were, for the most part, slight and transient. The thermometer at noon has not varied more than 4°; its medium height has been about 83°.

The ship's company have been three weeks, out of the nine, without fresh meat and vegetables.

The number of diseases entered on the sick list during the last month is considerably greater than that on any of the former; and this difference is entirely occasioned by the increase of small phlegmonous boils and slight accidents. The latter, I think, may be accounted for by a reference to the length of time the ship was in port, and the irregularities attendant thereon; for the former, which are prevalent to a very remarkable degree, I can assign no reason that seems to myself satisfactory.

The nine cases marked *fever* consisted of one pretty well marked mild remittent; three catarrhal or rheumatic fevers—that is, fevers obviously arising from suppressed perspiration, and characterised by superficial, rather than by visceral affections;—and five slight febrile accesses, terminating in a day or two under the use of depletion.

The bowel complaints were in general mild, and I have not met with any case of sufficient severity to entitle it to the name of dysentery.

The case marked cholera, and the three under the head of fish poison, were cases of extremely severe, though transient vomiting and purging; one following intoxication, the others produced by eating a crawfish. This is the only instance that has occurred at the Saintes of bad effects from fish, though this article of food was much used by the ship's company.

Of the two cases under the head erysipelas one was a febrile attack, accompanied by an inflammatory affection of the lymphatics of the lower extremity; the other was a common case of phlegmonous erysipelas affecting the whole of one of the arms, and terminating in sphacelus and death.

### Third Report—for August and September, 1814.

Of the period of two months, included in the above Return, the Venerable was six weeks at anchor in the harbour of the Saintes, a couple of days watering at Basseterre, Guadaloupe, and the rest at sea.

The weather, considering the season of the year, should, I imagine, be reckoned to have been good. Out of the sixty-one days, rain fell on twenty-four; and on ten of these in very great quantity, and accompanied by high winds:—the remaining days were in general fine. Fahrenheit's thermometer has varied from 80° to 87°; its medium altitude has been 82° or 83°. The *duty* of the ship's company has been, as usual, moderate; their diet good, and, for the most part, either fresh, or partly so. The interior of the ship has been constantly kept particularly clean and airy.

Of the six cases marked in the table fever, perhaps only two deserve that name. One of these (now under cure) is an inflammatory fever of considerable violence, and has been apparently got the better of by strong depletory means; the other was an old irregular intermittent. Three of the remaining four were true ephemeræ, as well in mildness as duration; the other was a mild case of *sun-stroke,* instantaneously removed by a copious bleeding.—And here, in illustration of the extreme healthiness of this station at present, and of the Saintes more particularly, I cannot avoid observing, that, although, during the whole period of our stay in port, thirty or forty men were permitted to go on shore one day in every week, and others occasionally; and although these, almost to a man, got intoxicated,—exposed themselves in this state to the sun's rays by day, and many of them to the night air;* yet not one of them was afterwards attacked with fever, nor with any other disease which could be attributed to these circumstances. As the island is hilly and dry, and contains only a very small portion of swampy ground, and this at

* Judging from my own observations during a residence of nearly four years, at different times, in the West Indies, I would say, in opposition to the general opinion, that *dews* are extremely slight in the Carribean sea and Leeward Islands. A heavy fall of dew I have never witnessed in any harbour, or in any of the islands; and at a considerable distance from land have never seen even a very slight one. I have often examined, during the night, the leaves and branches of the trees and shrubs, and do not recollect ever to have found these damp—much less wet. It is partly this feature of the *Indian night,*—or at least of the Carribean,—that gives to a moon-light walk in that climate a loveliness and a luxury which they only who have enjoyed its delights can fully appreciate.

O! in thy beams, thou lovely moon!
What heart e'er sigh'd for garish noon?
Mid scenes so bright, and air so bland,
What breast e'er long'd for northern land?

some distance from the places usually frequented by the seamen—ought this immunity from fever to be considered as any corroboration of the opinion that this disease, in warm climates, and in the warm seasons of cold climates, requires for its production something more specific than those causes—however violent—which are usually denominated *exciting causes*?

The case of Pneumonia, arising from an unknown cause, which proved fatal, was one of extreme violence, and remained constantly and completely unsubdued by the most copious venæsection. It was attended by some very anomalous symptoms, which are partly explained by the morbid appearances found on dissection, as detailed in the Journal.

Two of the cases of Ophthalmia were produced by the casual application of the juice of the manchineel-tree (Hippomane mancinella, Linn.) to the eye: they were slight, and easily cured.

Boils have been considerably less frequent. I know not why.

All the cases of *Fish-Poison*, except one, occurred at the Saintes. The particular species of fish that caused the affection was not ascertained. Out of about fourteen men who ate of it, five or six only were affected, and only three to such a degree as to be incapacitated for duty. The most marked symptoms were—excessive vomiting and purging, which came on, in general, about seven or eight hours after swallowing the fish,—head-ach,—slight inflammation of the fauces,—severe pains in different parts of the body,—uneasy pricking sensations over the whole surface, and burning heat in the palms and soles. These latter symptoms accompanied or followed the commencement of the vomiting and purging, but never preceded these. Copious dilution followed by opiates seemed to give most relief.

The two cases marked *Eruptions* were examples of the *Pompholyx benignus* of Dr. Willan; they occurred in boys, and were of short duration. Several others were seen.

The case of Hepatitis, the best marked of any I have seen originating in this country, was in the person of a drunkard; it has not been cured.

The four cases of Dysentery were chronic cases, which had been neglected in their early stage;* the others, of recent occurrence, were speedily cured, or, at least, soon got well.

---

* Two of these proved ultimately fatal at the hospital.

FOURTH REPORT — for November and December, 1814.

From the 1st of November to the 10th, and from the 18th of December to the 31st, the Venerable was anchored in Carlisle Bay, Barbadoes; during the intervening periods, she was employed in making passages, and in conveying troops, between that island and the colonies of Sainte Lucie, Martinique, Guadaloupe, and Antigua.— While on this duty, the ship was occasionally much crowded with people and baggage; and the crew were kept more than usually employed in mooring and unmooring ship, &c. &c.

The weather during the whole of November was fine and dry, generally with a good breeze more to the northward than during the preceding months: December has been rather showery, and altogether cooler and more squally and cloudy. The medium temperature of the last two months has been between 78° and 79° of Fahrenheit; but the change of season has been much more sensibly indicated by our feelings than by the thermometer.

Fresh beef and vegetables have been plentifully supplied.

The number of diseases entered on the sick-list has been less than in any two preceding months; and the diseases have not been severe in their character.

Of the six cases marked *Fever*, one was a Remittent sent on board from Guadaloupe in the last stage, and which proved fatal in Antigua hospital. Three others, which, in their commencement, exhibited marks of considerable violence, got speedily well under the use, chiefly, of very copious v. s. and purging.* These, with several other ephemeral affections, which rarely outlasted the operation of a powerful cathartic, were all the cases of fever that occurred among the crew of this ship; but three soldiers, who were sent on board labouring under this disease, died of it — one on board, and two at the hospital shortly after being landed. In these three cases, the disease had existed two whole days before I saw them, and was already evidently accompanied by so much organic

---

* To prevent misconception, I may mention, that by the expression "copious v. s." in these papers, I mean the detraction of 36, 40, or 45 oz. of blood on the first seizure, and the repetition of bleedings of 30, 20, 16 oz. after intervals of 4, 8, 12 hours, according to the severity and duration of the fever; so that within the first 48 or 50 hours (if the fever remained so long unsubdued), 2, 3, 4, 5, 6, 7 or 8 pounds of blood, according to circumstances, were taken away.

and structural derangement (in the brain and stomach), as to leave but feeble hopes of a favourable termination. Unchecked, or only momentarily relieved, by the means which seem to produce such marked amelioration in the very commencement of the fevers of this country (or, at least, in the fevers which have occurred in this ship), these cases proceeded to a fatal termination through the intermediate stages of precordial torture—universal yellowness—and black vomit.

The cases of *Flux* have been generally very mild.

The two cases marked *Hepatitis* were Phlegmasiæ, which had their seat in some viscus below the diaphragm, but whether in the liver or not I am not positive.

The case of *Nostalgia* occurred in an officer, and was, in the end, strongly marked by the general derangement of functions which it produced in the system.

The state of health of this ship is a pretty fair specimen of the general health of the navy on this station,—where fever (i. e. the violent endemic), dysentery, and ulcers, once so prevalent and fatal, may now be considered as rare diseases.

### Fifth Report — for January and February, 1815.

The weather, during the last two months, has had completely the winter character,—being, when compared with the summer season of this climate, boisterous, cold, cloudy, and unsettled. Passing showers fell almost every second day, but there were no continued heavy rains. The medium height of the thermometer has been between 77° and 78°.

Something more than one half of the period comprehended in this Report, the Venerable was cruising; the remaining time she lay in Carlisle Bay, Barbadoes. While in port, the ship's company were paid prize-money, and had frequent permission to go on shore;—consequently, they were not over and above temperate and regular in their habits.

Fresh beef and vegetables were served about one half the number of days the ship was in harbour, and, on the intermediate days, were always to be purchased; at least vegetables.

Of the diseases of these two months, the number is certainly considerably smaller than on any two months of last year: but whether this ought to be attributed to the people becoming more habituated to the climate,—to the season of the year,—or to any other accidental circumstance,—I am very undetermined.

The cases of fever were ephemeral attacks of very little consequence.

Under the title *Erysipelas* I have noted three smart febrile, accessions accompanied from the beginning with, and characterized by, a slight inflammation of the lymphatic glands on the upper and fore part of the thigh (similar to several cases before noticed), and a slight erysipelatous affection of the leg of the same side. These all got well in a few days under the use of purgatives only.

Under the name of *Colic* I have classed eight cases of very severe disease, about the real name and nature of which I am not quite decided. *Colica pictonum* I believe to be the name under which they would in general be classed: but they appeared to me to differ as much from this disease, as described by authors, as they did from the ordinary inflammations of the abdominal viscera. My treatment was strictly and strongly antiphlogistic and depletory. Under it the patients were soon freed from their pains and derangement of the alimentary canal; but great debility and low irregular fever came on (partly perhaps a consequence of the mode of treatment) which rendered it expedient to send four of them to the pure air and recruiting regimen of the hospital. They are now (March 1st) recovering.*

Two of the cases of flux have been tedious.

I think it is very probable, that most of the cases of colic were caused by intemperance; as, with two exceptions, all the men affected with these had been previously on shore, on leave, and had drunk much rum and porter: Several could distinctly trace the origin of their disease to the period of intoxication.

### Sixth Report—for March and April 1815.

With the exception of twelve or thirteen days in Car-

* They all perfectly recovered, and did not suffer any relapse during the twelvemonths they afterwards remained in the West Indies. In the early stages of all these cases, the blood drawn did not exhibit any of the usual marks deemed characteristic of inflammation; in the latter stage, when there were present a low irregular fever and *very great debility*, a few ounces of blood drawn from several of these patients (a mode of practice adopted partly by way of experiment, and which afforded very considerable and obvious relief) uniformly presented a very strong buffy coat.

lisle Bay, Barbadoes, the Venerable was employed all the month of March in cruising. On the 8th of April she went into English Harbour, Antigua, where she remained to the end of that month.

There has been nothing very particular in the general state of the weather. March was very fine and dry: in April there scarcely passed forty-eight hours without some showers; but there were heavy rains on two days only.

The atmospheric temperature has been gradually increasing with the sun's progress northward: the medium height of the thermometer in March was between 79° and 80°; in English Harbour, in April, it was, at least, 84°.

The diet has been as usual; fresh beef and vegetables having been served at least twice a-week while the ship was in port.

The duty of the ship's company up to the 8th of April was very light, with the exception of a few days in the end of March occupied in watering at Barbadoes; from the 8th of April to the end of the month the duty was more severe, the crew being employed in getting out and in two of the masts, and in otherwise refitting.

Until our arrival in English Harbour the diseases entered on the sick list were as few in number, and as mild in kind, as at any period of the ten preceding months; in fact, of all the acute diseases noted in the above Table, only four or five occurred before this period.

Three days after anchoring, the dysenteric and febrile affections began to make their appearance, and continued to occur, almost daily, during our stay.

In attempting to assign a cause for this—to us unusual though slight epidemic, (I speak both of the fever and dysentery, as I think they had both the same cause) I am inclined to give the principal, if not the whole share, to one or other of the following circumstances, or to both conjoined—viz. severe labour in the heat of the sun, and checked perspiration from exposure to sudden gusts of wind. I am led to adopt this opinion from the following considerations:—

1st. From the likelihood of the cause mentioned producing the effect in question;—particularly as, owing to the loftiness and steepness of the hills surrounding *Freeman's Bay* (where the ship lay) the wind came to us, not in an uniform stream, but only in heavy gusts after intervals of an oppressive calm. This was more remarkably the case during the first eight days. These gusts of wind were really disagreeably *cold*, and must have had considerable effect upon bodies exhausted by labour under a sun almost vertical, and bathed in general perspiration.

2d. From the character of the diseases themselves;—many of them being complicated with catarrhal symptoms, particularly the dysenteric cases.

3d. From the unusual prevalence of diseases generally allowed to depend on a disordered state of the perspiratory organs, e. g. rheumatism and catarrh;—many cases of the latter being observed that were not entered on the sick list.

4th. From there appearing no other unusual alteration in the state of external circumstances to which the alteration in the state of health could be attributed.

That it was not caused by exhalations from the land, seems probable from these two circumstances:—1st. All the land to windward of Freeman's Bay is high and dry, and contains none of that swampy soil supposed to be the principal source of unwholesome exhalations: And, 2ndly, of fifty men who were permitted to go on shore, and of whom the greater part remained upwards of twenty-four hours and had frequent occasion, in their researches after rum, to traverse the lowest and most marshy parts of the land in the neighbourhood,—only one was afterwards taken ill; and, in this patient, also, the fever was attended by some catarrhal symptoms, and did not make its appearance till sixteen days after his return on board.

That it did not spring from intemperance in the use of spirituous liquors, I think is evident; because, in the first place, owing to the great difficulty of procuring rum in English Harbour, there has been less intemperance among our people in this port than in other ports; and, in the second place, because, as has been already mentioned, only one case of fever occurred among those men who had been on shore, and who, there is little doubt, while on shore, made the most of their time in compensating for the lack of liquor on board.

With respect to the character of these febrile and dysenteric affections, which in all amounted only to twenty-six, it is obvious, I think, from their successful issue (they being all cured) that they were very mild in comparsion with those that were wont to desolate this harbour in former years. Although several of the fevers showed signs of considerable violence on the first attack, yet all of the patients were convalescent on the third day. The practice on board was to bleed largely immediately on the first attack, and to give strong and repeated purgatives; and the same mode of treatment, varying somewhat according to circumstances, with the addition of cold bathing when the heat was high, was followed at the hospital; only, that Dr. Crichton generally increased the proportion of calomel in his purgatives, so as occasionally to affect the mouth.

The dysenteries were equally easy of cure.

The case of colic resembled those mentioned in the last Report.

### Seventh Report—for May and June, 1815.

Of the period of time included in this Report, the Venerable was four days in English Harbour, Antigua; one at anchor off St. George, Grenada; nine in Carlisle Bay, Barbadoes; twelve in Gros Islet Bay, St. Lucie; eleven at Fort Royal and St. Pierre, Martinique; and the remaining days at sea.

The rainy character of April has been retained, and indeed increased, during the two last months; rain having fallen on thirty days of the sixty-one, and on twenty of these the rains were very heavy, and accompanied by squalls and intervening calms. The following are the thermometrical results:—

| | Highest. | Lowest. | Mean. |
|---|---|---|---|
| May | 86° | 78° | 83° |
| June | 84° | 80° | 82° |

The diet has been much the same as usual; fresh beef and vegetables having been served on nearly one-fourth of the time.

There has been nothing very particular in the duties of the ship's company, nor in the general economy of the ship: it is, however, proper to observe, that the ship was a good deal crowded for the space of a week in the end of May and beginning of June by troops we had on board; and, also, that, on account of the frequent rains, not only the mens' persons have been subjected to an uncommon degree of moisture, but the interior of the ship also has been necessarily less dry, clean, and airy than has been customary.

Of the cases of fever noted in the Table, five were the product of English Harbour, and exactly resembled those described in my last Report. These, as also all the others, the greater number of which were mere ephemeræ, were speedily cured, as were also the cases of flux.

The case of erysipelas resembled those formerly described as affecting the lymphatics of the lower extremity.

Consumption continues to be, comparatively speaking, of frequent occurrence, and of most untractable character: a permanent alleviation of symptoms has been rarely attained, and no cure has been effected in this ship, nor, I believe, at the hospital. Perhaps the fatal case of pneumonia, noticed in the Table, ought to be considered as one of phthisis, inasmuch as,—although the affection which immediately occasioned death (in the space of three days) was certainly inflammation of the lungs,—as proved by dissection,—yet this patient had laboured under cough and copious expectoration for many months previously. This is the *second Purser's Steward* that has fallen a victim to pulmonic disease since the arrival of the Venerable in this country; and, although in both cases there certainly existed another exciting cause, (intemperance) yet I have no doubt that the peculiarity of their duties and of the atmosphere in which they lived, was the principal source of their disease.* On this occasion I cannot avoid remarking, that ships fitted with proper ventilators,—particularly that kind of ventilators in which the circulation of the air is promoted by the use of fires,—must, in warm climates, possess very superior advantages in the purity of the air of the orlop and hold, over those ships which are without them. I am sorry to say, that the Venerable is of this latter description..

EIGHTH REPORT—for July and August, 1815.

Of the sixty-two days comprehended in the above Table, the Venerable was stationed twenty-three in Carlisle Bay, Barbadoes, twenty-four in the harbour of the Saintes, and fifteen at sea, or at anchor for a very short time off different islands.

July and August have been rainy months: Showers fell on one half of the days of the former, and on two thirds of the latter month; and on some of these the rains were very heavy. The following rate of atmospheric heat has been shown by the thermometer:—

| | Greatest. | Least. | Mean. |
|---|---|---|---|
| July | 84° | 79° | 82° |
| August | 88° | 79° | 83° |

* It may be, perhaps, proper here to remark, for the sake of those of our readers unacquainted with the navy, that the Purser's Steward almost constantly lives in the cock-pit—that is, in the *hold* or lower part of the ship—where the air is very impure from the admixture of foreign matters and the want of frequent renewal. EDIT.

Fresh beef and vegetables have been served about one-fourth of the time.

The duty of the ship's company has, perhaps, been more than usually severe, particularly in August, on account of the capture, by our naval and military forces, of the island of Guadaloupe, which was effected in the beginning of this month. In the embarkation and debarkation of troops, stores, &c. the men have been much exposed in boat-service; and, in the latter part of August, a good many of them have been away from the ship in detained vessels, &c.

The internal economy has been the same as usual: owing to the frequent rains, the decks have perhaps been wetter, and, for a couple of weeks, they were a good deal crowded by troops and baggage.

The number of diseases entered on the sick-list, during the last two months, exceeds considerably that of any two preceding, except the corresponding months of last year,* The present number (one hundred) is the same as that of 1814, and agrees with it also in being half-made-up of slight wounds and trifling inflammations.

Fever has been much less frequent than usual, and the cases that occurred have been of less violence than at any former period. Blood-letting did not appear so necessary, —at least it has been more sparingly employed.

Visceral diseases have, however, been both more frequent and more violent. Of the thirteen cases marked *flux*, several have been well-marked and violent dysenteries; one of which—that was sent to the hospital—proved fatal. This is the only death from this disease that has occurred in the crew of this ship since her arrival in the West Indies. Under the title *hepatitis*, are included those inflammatory diseases which had their site in some part of the abdomen other than the intestinal canal. Perhaps only three of these were true inflammations of the liver; they have been, however, all rather severe and obstinate.

Phthisis has been, as usual, frequent, and as usual, most untractable.

The cases marked *colic* would, perhaps, better be named *painful constipation*; of course, they were very slight.

*P. S.* The Venerable remained in the West Indies until March 1816; and, with the exception of thirty-three

---

* I now perceive this to be a mistake; April and May exhibit a greater number. The mistake arose from comparing *only* the different *two months* that were reported on *together*.

cases of fever, which occurred in September and October, (the produce of the swamps of Point-a-Pitre, Guadaloupe) preserved a state of health very similar to that recorded in the above Reports. Of these thirty-three cases of fever, two proved fatal; and, when we consider the uniform recovery of those treated in *the commencement* of this disease, it is, perhaps, probable, that the circumstance of these two cases not having been seen till the evening of the second day, may have contributed considerably to the unfavourable result. J. F.

*Penzance, Dec.* 18, 1817.

